# Disentangling substance use and related problems: urgency predicts substance-related problems beyond the degree of use

**DOI:** 10.1186/s12888-021-03240-z

**Published:** 2021-05-07

**Authors:** Malin K. Hildebrandt, Raoul Dieterich, Tanja Endrass

**Affiliations:** Institute of Clinical Psychology and Psychotherapy, Chair of Addiction Research, Faculty of Psychology, Technische Universität Dresden, Chemnitzer Str. 46a, 01187 Dresden, Germany

**Keywords:** Substance use, Impulsivity, Sensation seeking, Dimensional, Urgency

## Abstract

**Background:**

Substance use disorders are reliably associated with high impulsivity and sensation seeking. Importantly, both precede problematic substance use, implicating them as risk factors. Individuals with substance use disorders show variable degrees of substance use (combined quantity and frequency) and substance-related problems and differ in both aspects from healthy controls. Dimensional research has indicated differential associations of impulsivity-related traits as well as sensation seeking with the degree of substance use and substance-related problems. The current study aimed to clarify whether impulsivity-related traits and sensation seeking predict substance-related problems above and beyond the degree of substance use and are thus specifically linked to problems, the dimension that characterizes substance use disorders.

**Method:**

We assessed impulsivity-related traits and sensation seeking using self-report, as well as delay discounting, a behavioral indicator of impulsivity, in a sample of 258 substance-using adults.

**Results:**

Sensation seeking and impulsivity-related traits significantly predicted the degree of substance use, with sensation seeking explaining the largest portion of variance. In contrast, self-reported impulsivity, in particular when experiencing negative emotions (urgency), but not sensation seeking or delay discounting, predicted substance-related problems when controlling for the degree of substance use.

**Conclusions:**

This suggests that urgency, but not sensation seeking, may be specifically linked to substance-related problems and thus especially relevant for substance use disorders. Taken together, this study underlines the necessity to assess and control for the degree of substance use in risk factor research concerning substance-related problems. Thus, it may inform future research improving targeted prevention and therapy.

**Supplementary Information:**

The online version contains supplementary material available at 10.1186/s12888-021-03240-z.

## Background

Substance use disorders (SUDs) are characterized not only by substance use itself but by problems related to substance use [1], such as the inability to control substance intake, or the failure to comply with social responsibilities. Not all frequent substance users develop these problems to the same extent. It is crucial for targeted prevention and treatment to identify factors that specifically influence the development of problems. Most of the literature on SUDs relies on comparisons of individuals diagnosed with SUDs and healthy controls in order to do so. Here, the patients differ from healthy controls in both the degree of substance use (quantity and frequency) and substance-related problems. This hinders evaluating whether the examined potential risk factors are truly associated with the development of substance-related problems, the dimension that is critical for the diagnosis of SUDs. The specific associations of potential risk factors with the degree of substance use and substance-related problems remain unclear. Here, we are proposing a dimensional approach examining the associations of potential risk factors with substance-related problems while statistically controlling for the degree of substance use. Consequently, we assessed both variables separately and dimensionally in a population of substance users of varying degrees of substance use. Using this approach, we aimed to take a first step in re-evaluating the role of impulsivity-related traits as risk factors for SUDs. High levels of impulsivity-related traits are robustly found in individuals with SUDs [2–5]. So far, it has never been tested whether this is driven by the association of these factors with the degree of substance use or whether they are, beyond that, incrementally relevant for substance-related problems across different substances. Particularly, controlling for the degree of use has been neglected in previous work.

Overall, impulsivity is robustly associated with SUDs (for a review see e.g. [6]). However, in the relevant literature, the conceptualization of impulsivity is not always coherent and hence impulsivity may be best described as an umbrella term subsuming several related traits. In an attempt to solve this empirically, Whiteside and Lynam [7] were the first to conduct a factor analysis of various questionnaires assessing impulsivity. This analysis and similar factor analyses repeatedly suggested that impulsivity may be divided into four distinct, yet related traits which constitute the UPPS model of impulsivity: Urgency, (lack of) Premeditation, (lack of) Perseverance and Sensation seeking [7, 8]. The first three traits conform with a narrow definition of impulsivity, understood as the propensity to act quickly while disregarding long-term negative consequences [9] and we will therefore subsume them as impulsivity-related traits. More specifically, urgency describes the tendency to act rashly when experiencing negative emotions, (lack of) premeditation describes the tendency to act without thinking, and (lack of) perseverance describes the tendency not to finish tasks [10]. Sensation seeking does not conform with this narrow definition of impulsivity and has recently been pinpointed as a separate construct [11] that correlates only weakly with impulsivity-related traits (between r = 0.00 and r = 0.18 [7, 12];), suggesting that the tendency to seek excitement is to a large extent independent of the tendency to act rashly. The UPPS model is useful for a comprehensive assessment of impulsivity as it builds on several conceptualizations of impulsivity from the literature and other conceptualizations can be allocated to the UPPS traits (see e.g. [13]). For example, regarding the widely used Barratt Impulsiveness Scale [14], the “lack of planning” and “attentional impulsivity” subscales can be allocated to the traits (lack of) premeditation and urgency, respectively.

Longitudinal studies suggest that sensation seeking prospectively predicts the risk to develop SUDs (e.g .[4]). Further studies indicate impulsivity-related traits as risk factor for SUDs by showing that impulsivity-related traits prospectively predict the degree of substance use as well as related problems with similar [15, 16] or higher [17] associations of impulsivity-related traits with problems than with the degree of substance use. Notably, substance use and problems do not prospectively predict impulsivity-related traits [18]. One study provides first evidence that a composite score that included impulsivity-related measures, sensation seeking and emotional control prospectively predicts substance-related problems even when controlling for substance use (with a medium effect size [16];).

Research further suggests that impulsivity-related traits as well as sensation seeking are differentially related to the degree of substance use and problems. Sensation seeking and premeditation are associated with the degree of substance use [8, 19, 20]. A meta-analysis that assigned various impulsivity measures to the traits of the UPPS model found that all traits were similarly related to the degree of use, while urgency was the trait most strongly related to problems [13]. The trait of urgency, specifically when experiencing negative emotions, as it was conceptualized in the UPPS model, has also been linked to other types of problem behavior (e.g. bulimic symptoms, problematic gambling, self-injury and sexual risk taking [21–23];). Moreover, although the evidence is so far restricted to alcohol use, urgency and perseverance were shown to be associated with problems, even when controlling for the degree of substance use [20]. Importantly, the association of sensation seeking with problems disappeared when the degree of substance use was statistically controlled for. These findings underline that the approach we propose here is suitable for identifying factors specifically associated with substance-related problems.

One widely studied behavioral indicator of impulsivity is delay discounting, the subjective devaluation of rewards based on how far in the future they become available [24]. This index of the preference for smaller sooner over larger later rewards is of high conceptual relevance to SUD research, as it closely resembles behavior observed in individuals with SUDs, i.e., choosing immediate drug effects over long-term goals (e.g., taking care of family or school/job etc.). Empirically, higher delay discounting in individuals with SUDs [25] and associations of delay discounting with substance use frequency [26] and SUD severity (indicating substance-related problems) are consistently observed [27–29]. Delay discounting correlates with premeditation [19, 30–32], but also urgency [30] and sensation seeking [32], but a meta-analysis suggests that these associations are overall rather weak [32].

The goal of this study was to extend the finding that impulsivity-related traits, but not sensation seeking, predict alcohol-related problems above and beyond the degree of substance use [20]. We investigated this relationship in an online survey in a sample of undiagnosed (poly-)substance users with widely varying degrees of substance use. Thereby, we aimed to examine if impulsivity-related traits, but not sensation seeking, may be specifically linked to substance-related problems (and thus truly be relevant for SUDs) across different substances. Furthermore, we assessed delay discounting to examine whether self-reported and behavioral impulsivity-related traits distinctly relate to substance use variables. We expected that impulsivity-related traits (self-report and delay discounting) as well as sensation seeking would predict the degree of substance use, while only impulsivity-related traits (more specifically urgency, perseverance and delay discounting), but not sensation seeking, would predict substance-related problems when controlling for the degree of substance use.

## Method

### Participants

Participants were recruited mainly through advertisements in clubs, webpages associated with the techno music scene, and counseling centers as well as through postings and flyers. Inclusion criteria were assessed as self-report questions in the online survey and were: (1) current use of at least one substance at least once a month, (2) age between 18 and 40 years, (3) native German speakers, (4) no reported use of any substance (except for nicotine) on the day of the survey. We excluded these participants to avoid effects of acute intoxication on questionnaire responses. The final sample, after excluding those survey participants who did not fulfill the inclusion criteria (*N* = 114) consisted of 258 participants.

**Fig. 1:**
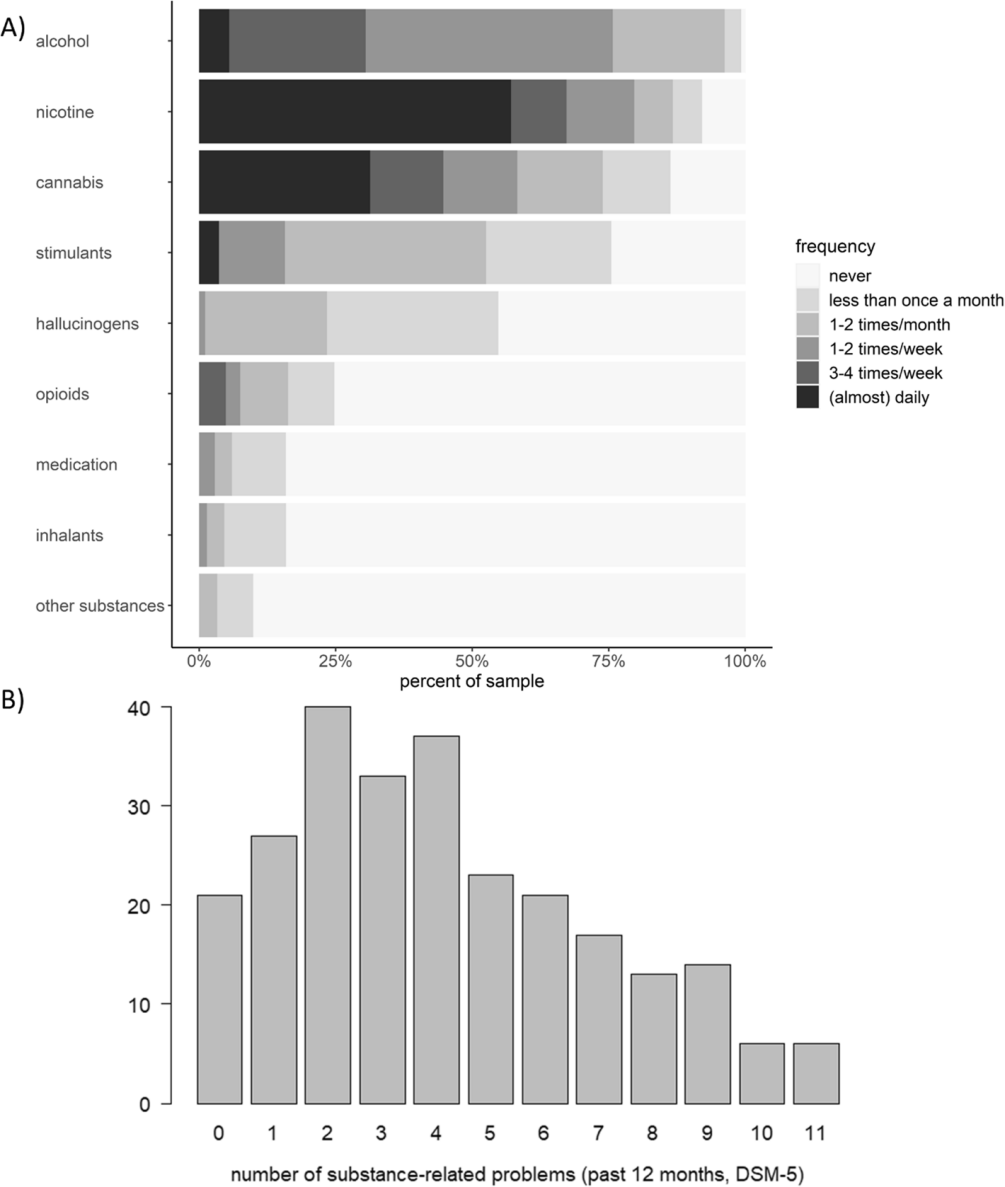
Descriptive information on substance use and substance-related problems**.** *Note.* **a**. Distribution of substance use frequency in percent separately for each substance class within the current sample. **b**. Histogram of substance-related problems: the y-axis depicts the number of participants reporting the respective cumulative number of self-reported substance-related problems (DSM-5 criteria, irrespective of specific substance). Note that substance-related problems were assessed across substances and thus ≥2 problems do not entail an SUD diagnosis

Participants reported a wide range of substance use frequency, quantity and related problems. As this was an online survey, we did not confirm SUD criteria by a diagnostic interview. Hence, the current sample is a non-clinical and undiagnosed sample, but likely includes individuals at risk for SUD diagnosis as indicated by the range of problems participants reported (see Fig. 1). Moreover, 54.7% reported frequent use (at least three to four times a week) of at least one substance. See Fig. 1 and Table 1 for sociodemographic and substance use-related characteristics of participants. Further details are available in the supplementary material.

**Table 1:** Sociodemographic and substance use-related characteristics of participants

Characteristic			*N*	*%*
**Gender**				
female			148	57.4
male			109	42.2
not specified			1	0.4
**Secondary school degree**				
upper			226	87.6
intermediate			28	10.9
lower			2	0.8
**Current use of**				
alcohol			236	91.5
nicotine			159	61.6
cannabis			117	45.3
stimulants			79	30.6
hallucinogenic substances			29	11.2
opioids			14	5.4
misused medication			5	1.9
inhalants			4	1.6
other substances			3	1.2
	M	MD	SD	range
age	26.1	25.0	5.0	18–40
currently used substance classes	2.5	2.0	1.3	1–6
substance classes used lifetime	4.7	5.0	2.0	1–9
total degree of substance use	35.9	36.5	19.4	3–89
substance-related problems	4.2	4	2.9	0–11

*Note. M, MD,* and *SD* represent mean, median, and standard deviation, respectively. Note that substance-related problems were assessed across substances and thus ≥2 problems do not entail an SUD diagnosis

Participants gave informed consent prior to participation and could earn course credit (six participants) or take part in a lottery to win one of three 10 € vouchers. The study protocol was approved by the local ethics committee (EK 146042019) and followed the guidelines stated by the Declaration of Helsinki.

### Procedure

Participants completed an online-survey (approx. 20 min) comprising sociodemographic information, inclusion criteria, and the measures described below.

### Material

#### Substance use questionnaire

This questionnaire measured the frequency and quantity of substance use for the following substance classes from the DSM-5: alcohol, tobacco, cannabis, stimulants, opioids, hallucinogenic substances, misused medication, inhalants, other substances. Participants were first asked whether they had ever used any of the substances from the respective substance class. If they answered ‘yes’, participants indicated, among other variables not relevant here, their average frequency of use within the past three months on a 6-point Likert scale ranging from 1 to 6 (*almost daily*, *3–4 times a week, 1–2 times a week, 1–2 times a month, less than once a month, never*) and the average subjective quantity of use on a standard occasion within the past three months on a 5-point Likert scale ranging from 1 (*very little*) to 5 (*very much*). We computed individual degree of substance use scores for each substance class (product of quantity and frequency values, possible maximum: 30) as an intermediate step and summed these scores to compute the total degree of substance use score which was used in the analyses (possible maximum: 270).

#### Substance-related problems questionnaire

This questionnaire measured substance-related problems operationalized by the number of DSM-5 SUD symptoms from the A criterion participants reported to have experienced within the past 12 months (range 0–11). Questions were based on the German version of the Structured Clinical Interview for DSM-5 Disorders – Clinician Version [33] and adapted to the online application. Participants were instructed to consider all substances when judging whether they had experienced each symptom. Thus, this index does not equal a diagnosis of a specific substance use disorder, but rather an index of problem severity across substances as experienced symptoms may be attributable to different substances.

#### UPPS impulsive behavior scale

This 45-item self-report questionnaire assesses four subscales representing facets of impulsivity as identified by Whiteside and Lynam [10]: [1] negative urgency (e.g., “In the heat of an argument, I will often say things that I later regret.”; possible range: 12–48), [2] (lack of) premeditation (e.g., “I usually make up my mind through careful reasoning.”; possible range: 11–44), [3] (lack of) perseverance (e.g., “Once I start a project, I almost always finish it.”; possible range: 10–40), and [4] sensation seeking (e.g., “I welcome new and exciting experiences and sensations, even if they are a little frightening and unconventional.”; possible range: 11–44). Items are rated on a 4-point Likert scale ranging from 1 (*strongly agree*) to 4 (*strongly disagree*). If necessary, items are reverse coded so that higher values on a subscale always indicate higher impulsivity/sensation seeking. The subscales of the German version [34] have good internal consistency (cronbachs α = .81–.85) and external validity [35].

#### Monetary choice questionnaire (MCQ)

The MCQ (German version [36];) assesses delay discounting. It comprises 27 binary choices between varying amounts of money (8–66 Euros) available either now or with a delay (7–238 days). Values and delays of the options are predefined to cover a high range of discounting rates for low, medium, and high values of delayed rewards, respectively. The discounting rate *k* can be modeled assuming a hyperbolic function [37].

1 $$ V=\frac{A}{1+ kD} $$

where *V* is the subjective value of a delayed reward with the amount *A* after the delay *D*.

The parameter *k* is estimated based on the proportion of choices that are consistent with every possible value of *k* (for a thorouh description please refer to [38]). As *k* is dependent on value [39], *k* is estimated for small, medium and large delayed rewards, respectively, and the geometric mean of these values constitutes an individual’s *k*-value.

### Data analysis

All analyses were conducted in R [40]. We log-transformed the *k*-parameter due to skewedness. Pearson’s correlations were computed to examine bivariate associations. Two-tailed tests of the difference between two dependent correlations with one variable in common were conducted to test whether correlations differed significantly. Subsequently, we computed partial correlations of all predictor variables with substance-related problems controlled for the degree of substance use to explore their respective contribution outside the context of other predictor variables. Last, we conducted two hierarchical linear regressions for the degree of substance use and substance-related problems to identify the unique variance explained by sensation seeking, impulsivity-related traits, and log(*k*). For both regression models, we entered gender as a covariate in the first step, because it was significantly related to both outcome variables, while age was not (ps > .05). We excluded the group “gender not specified” from these regression analyses (*n* = 1). For the model predicting the degree of substance use, we added the UPPS subscales in the second and log(*k*) in the third step. For the model predicting substance-related problems, we added the degree of substance use as a covariate in the second step, the UPPS subscales in the third step and log(*k*) in the fourth step. To test on an exploratory basis whether a regressor explained more variance in the criterion variable than another regressor, we computed finite-sample F-statistics using the “linearHypothesis” function from the car package [41]. We applied a significance level of α = .05 for all analyses.

## Results

### Bivariate correlations

Discounting rates ranged from 0.000158 to 0.24853, with some choosing exclusively the smaller sooner or the larger later option, and were not significantly associated with any of the UPPS subscales (all *p*s > .05), except for a small positive correlation with urgency (see Table 2 for an overview of correlations). Delay discounting and (lack of) premeditation were significantly, but weakly, correlated with both the degree of substance use and problems. (Lack of) perseverance displayed stronger correlations (small to moderate) with both substance use variables. Sensation seeking correlated significantly stronger with the degree of substance use than with problems, r = .38 vs. r = .23, *t*(256) = 2.72, *p* = .01. In turn, urgency correlated significantly stronger with substance-related problems than with the degree of substance use, r = .33, vs. r = .18, *t*(256) = 2.67, *p* = .01. The degree of substance use and substance-related problems were strongly correlated.

**Table 2:** Means, Standard Deviations, and Correlations with Confidence Intervals for UPPS subscales, Delay Discounting (log(k)), the Degree of Substance Use and Substance-Related Problems

	Variable	*M*	*SD*	1	2	3	4	5	6
**UPPS**									
1.	Urgency	26.77	5.96						
2.	Premeditation	23.59	4.50	.32**					
				[.20, .42]					
3.	Persistence	20.40	4.41	.40**	.26**				
				[.29, .50]	[.14, .37]				
4.	Sensation Seeking	32.00	7.26	−.00	.32**	.01			
				[−.12, .12]	[.21, .43]	[−.12, .13]			
**MCQ**									
5.	log (*k*)	−2.28	0.75	.14*	−.00	.04	−.01		
				[.01, .25]	[−.12, .12]	[−.08, .16]	[−.14, .11]		
**Substance use variables**									
6.	Degree of use	35.89	19.36	.18**	.16*	.22**	.38**	.18**	
				[.05, .29]	[.04, .28]	[.10, .34]	[.27, .48]	[.06, .30]	
7.	Problems	4.15	2.85	.33**	.13*	.29**	.23**	.15*	.55**
				[.22, .43]	[.01, .25]	[.17, .39]	[.11, .35]	[.03, .27]	[.46, .63]

*Note. M* and *SD* represent mean and standard deviation, respectively. Values in square brackets indicate the 95% confidence interval. *UPPS* Urgency Premeditation Perseverance Sensation Seeking Impulsive Behavior Scale, *MCQ* Monetary Choice Questionnaire, *k* = delay discounting. * *p* < .05. ** *p* < .01

### Partial correlations

When controlling for the degree of substance use, urgency (*r*(256) = .28, *p* < .001) and perseverance (*r*(256) = .20, *p* = .001) significantly correlated with substance-related problems, while premeditation, sensation seeking and log(*k*) did not (all *p*s > .05).

### Relative contributions of UPPS subscales and delay discounting to substance use outcomes

#### The degree of substance use

In the first model, we regressed the degree of substance use on the UPPS subscales and log(*k*) while controlling for gender (see Table 3). The hierarchical multiple regression revealed that all UPPS subscales (except for premeditation) as well as log(*k*) explained significant unique variance in the degree of substance use, such that higher levels of urgency, perseverance, sensation seeking and delay discounting were associated with more substance use. In the complete model, sensation seeking had a significantly stronger effect on the degree of substance use than all other impulsivity-related predictors respectively, all *F*s > 4.13, all *p*s < .05. The complete model accounted for 25% of the variance in the degree of substance use.

**Table 3:** Hierarchical Multiple Regression Analyses with the UPPS Subscales and Delay Discounting predicting the Degree of Substance Use and Substance-Related Problems

Predictors	Degree of substance use					Substance-related problems				
	ΔR^2^	F	B	SE	β	ΔR^2^	F	B	SE	β
*Step 1*	.087**	24.45				.017*	4.432	4		
Male gender			11.57	2.34	.30**			0.75	0.35	.13*
*Step 2*						.305**	114.22			
Male gender								−0.23	0.31	−.04
Degree of substance use								0.08	0.01	.58**
*Step 3 - UPPS*	.144**	11.70				.068**	6.99			
Male gender			7.38	2.52	.19**			−0.06	0.33	−.01
Degree of substance use								0.07	0.01	.49**
Urgency			0.52	0.21	.16*			0.11	0.03	.23**
Premeditation			−0.09	0.28	−.02			−0.04	0.04	−.06
Perseverance			0.64	0.28	.15*			0.06	0.04	.10
Sensation seeking			0.83	0.18	.31**			0.03	0.02	.07
*Step 4 - MCQ*	.022**	7.26				.001	0.30			
Male gender			6.40	2.51	.16*			−0.08	0.34	−.01
Degree of substance use								0.07	0.01	.48**
Urgency			0.43	0.21	.13*			0.11	0.03	.22**
Premeditation			−0.08	0.27	−.02			−0.04	0.04	−.06
Perseverance			0.67	0.27	.15*			0.07	0.04	.10
Sensation seeking			0.86	0.17	.32**			0.03	0.02	.08
Log(*k*)			3.88	1.44	.15**			0.10	0.19	.03
Total R^2^	.253**	14.08				.391**	22.83			

*Note. ΔR*^*2*^ = change in proportion of explained variance; B = unstandardized estimate; SE = standard error of B, β = standardized estimate. * *p* < .05. ** *p* < .01

#### Substance-related problems

In the second model, we regressed substance-related problems on the UPPS subscales and log(*k*) while controlling for age and the degree of substance use (see Table 3). The hierarchical multiple regression revealed that the UPPS urgency subscale explained significant unique variance in substance-related problems, such that higher levels of urgency were associated with more problems above and beyond the degree of substance use, which also predicted substance-related problems. None of the other predictors were significantly associated with substance-related problems. In the complete model, urgency had a significantly stronger effect on substance-related problems than premeditation and log(k) (all *F*s > 5.57, all *p*s > .05) and sensation seeking, but this difference was only a marginally significant (*F* = 2.96, *p* = .09). The complete model accounted for 39% of the variance in substance-related problems.

## Discussion

This study examined the differential associations of impulsivity-related traits, sensation seeking, and delay discounting with the degree of substance use (combined quantity and frequency) and substance-related problems in a sample of (poly-)substance users. Thereby, we aimed to disentangle which of these potential risk factors were associated with the degree of substance use and which ones were specifically relevant for substance-related problems and thus for the diagnosis of SUDs. As expected, sensation seeking was the strongest predictor for the degree of use and the degree of use was the strongest predictor for substance-related problems. Importantly, although the degree of use explains a large part of the variance in substance-related problems, impulsivity-related traits did explain variance in substance-related problems above and beyond the degree of use.

First, sensation seeking, self-reported urgency and perseverance, as well as delay discounting, explained unique variance in the degree of substance use. Sensation seeking was the strongest predictor, explaining significantly more variance in the degree of substance use than all other predictors. This conforms with the reviewed literature [8, 20, 42] and indicates that high expressions of both impulsivity and sensation seeking independently, and more so combined, may increase the risk to engage in pronounced substance use. This may already be harmful, e.g. in terms of bodily consequences, but does not necessarily go along with substance-related problems.

Second, the degree of substance use explained about 30% of the variance in substance-related problems, underlining the undisputed substantial relevance of use patterns for SUDs [43]. Although such a large part of variance was explained by the degree of use, including the UPPS traits into the model added significant explanatory value (7% of total variance in substance-related problems). This was primarily due to the impulsivity-related trait of negative urgency which explained significant unique variance in substance-related problems above and beyond the degree of substance use, while sensation seeking did not. This finding is in line with prior research [20] but extends the evidence beyond alcohol use and shows that urgency is specifically linked to substance-related problems, the dimension critical for SUDs, even when controlling for substance use and across substances. Building on evidence showing that high levels of urgency precede problematic substance use (e.g. 42), this finding underlines the role of urgency as a potential general risk factor for substance-related problems. Furthermore, this is the first study to combine self-report with behavioral measures of impulsivity using the approach to SUD risk factor research we propose here. This highlights the special role of urgency for SUDs, as this trait was associated with problems even when controlling for the degree of use as well as behavioral impulsivity (delay discounting) and other impulsivity-related traits.

Delay discounting, a behavioral measure of impulsivity, was weakly associated with both the degree of substance use and problems, but the association with problems disappeared when controlling for the degree of use. This is not surprising, considering that delay discounting was only weakly related to substance-related problems in the first place, closely resembling the effect size found in a meta-analysis [27]. This finding indicates that the consistent associations of delay discounting and SUDs described in the literature may be best explained by underlying and confounding differences in the degree of substance use. Although a high degree of use may be inherently harmful, this contradicts the role of delay discounting as a specific risk factor for substance-related problems and thus SUDs discussed in the literature (e.g. 3). However, as this is the first study to examine associations of delay discounting with substance-related problems controlling for the degree of use, this finding needs replication before drawing final conclusions.

### Limitations and future directions

The dimensional approach we applied in this study proved suitable for identifying differential associations of potential SUD risk factors with substance-related problems controlling for the degree of use. Nonetheless, it requires a longitudinal approach to test whether these factors truly constitute specific risk factors for developing substance-related problems. Furthermore, the delay discounting questionnaire we used in this study uses relatively low monetary rewards. Research suggests that high monetary offers better distinguish substance users from controls [44]. Hence, tasks using higher monetary offers may be more suitable for substance-using populations and delay discounting assessed with such tasks may show a specific association with substance-related problems. Importantly, our sample was predominantly highly educated. It is possible that this acted as a protective factor buffering effects of e.g. delay discounting on substance-related problems. Thus, further studies including more representative samples are warranted to consolidate the generalizability of the associations described in this study. Due to temporal restraints in the online-survey, we did not assess substance-related problems separately for each substance or substance class. Therefore, we could not directly test whether the examined associations differed between substances but rather demonstrated that they were overall significant when all substances were combined. Also, the assessment of substance-related problems was purely based on self-report, while clinical interviews may provide a more nuanced and comparable picture. For reasons of brevity of the online survey and our decision to combine individual substances to substance classes, we did not assess objective quantities of substance use. The results of this study do not change when using only total frequency rather than the total degree of substance use score as a control variable (see supplementary material for further details). Future studies may include positive urgency, as this facet shows unique associations with substance use outcomes [21] and may thus contribute additional insights.

## Conclusions

This study showed that the impulsivity-related traits of urgency and to some extent perseverance, but not sensation seeking, were specifically linked to substance-related problems, the dimension that is crucial for SUDs. We applied a dimensional approach to SUD risk factor research examining the associations of potential risk factors with substance-related problems while statistically controlling for the degree of substance use. The present study demonstrated that this approach is suitable for identifying factors specifically linked to substance-related problems and thus SUDs. Hence, future longitudinal studies using this approach may clarify the role of impulsivity-related traits, alongside others, as risk factors for SUDs. To identify risk factors specifically linked to the development of substance-related problems, rather than predominantly a high degree of use, would convey important implications for practice. A focus on these factors may help to identify individuals within substance using populations who are more in need for preventive measures in order to avoid a transition to problematic substance use. However, longitudinal studies are needed to substantiate the current finding. Furthermore, they may be relevant target points for therapeutic interventions. Taken together, the approach applied in this study may pose a starting point for further research advancing targeted prevention and therapy.

## Supplementary Information

**Additional file 1: Table S1.** Means, Standard Deviations, and Correlations with Confidence Intervals for UPPS subscales, Delay Discounting (log(k)), Substance Use Frequency and Substance-Related Problems. **Table S2.** Hierarchical Multiple Regression Analyses with the UPPS Subscales and Delay Discounting Predicting Substance Use Frequency and Substance-Related Problems.

## Data Availability

The dataset supporting the conclusions of this article is available in the OSF repository, DOI 10.17605/OSF.IO/CWNRG, https://mfr.de-1.osf.io/render?url=https://osf.io/8wgts/?direct%26mode=render%26action=download%26mode=render.
