# Supplementary material for: Disentangling substance use and related problems: urgency predicts substance-related problems beyond the degree of use
Source: BMC Psychiatry. 2021 May 7;21:242. doi: 10.1186/s12888-021-03240-z (PMC8103599; doi:10.1186/s12888-021-03240-z)
Supplement: Supplementary file 1 — Additional file 1: Table S1. Means, Standard Deviations, and Correlations with Confidence Intervals for UPPS subscales, Delay Discounting (log(k)), Substance Use Frequency and Substance-Related Problems. Table S2. Hierarchical Multiple Regression Analyses with the UPPS Subscales and Delay Discounting Predicting Substance Use Frequency and Substance-Related Problems. [file 12888_2021_3240_MOESM1_ESM.docx]

#

Disentangling Substance Use and Related Problems: Urgency Predicts Substance-Related Problems Beyond the Degree of Use.

Malin K. Hildebrandt^a^, Dr. Raoul Dieterich^a^, Prof. Dr. Tanja Endrass^a^

^a^Institute of Clinical Psychology and Psychotherapy, Chair of Addiction Research, Faculty of Psychology, Technische Universität Dresden, Chemnitzer Str. 46a, 01187 Dresden, Germany

Corresponding author:

Hildebrandt, Malin K.

[malin.hildebrandt@tu-dresden.de](mailto:malin.hildebrandt@tu-dresden.de)

Tel.+49 351 463-38579

Fax+49 351 463-36984

E-Mail of co-authors:

[raoul.dieterich@tu-dresden.de](mailto:raoul.dieterich@tu-dresden.de)

[tanja.endrass@tu-dresden.de](mailto:tanja.endrass@tu-dresden.de)

**Supplementary Material**

**1. Method – Participants**

Participants with upper and intermediate degrees did not differ significantly on any of the variables relevant for our analyses (all *p*s > .05), except for higher delay discounting in participants with intermediate degrees (log(*k*); *t*(252) = 3.52, *p* < .001). Due to the skewed distribution of school education, we did not compute comparisons with the lower degree group. Age was not significantly associated with any of the variables relevant for our analyses (all *p*s > .05).

**2. Results**

Here, we provide the results of the analyses using total frequency (sum of all substance-specific frequency scores) rather than the total degree of substance use score.

**2.1 Bivariate correlations**

**Supplementary Table S 1**

*Means, Standard Deviations, and Correlations with Confidence Intervals for UPPS subscales, Delay Discounting (log(k)), Substance Use Frequency and Substance-Related Problems*

|  |  | *M* | *SD* | 1 | 2 | 3 | 4 | 5 | 6 |
| --- | --- | --- | --- | --- | --- | --- | --- | --- | --- |
|  | **UPPS** |  |  |  |  |  |  |  |  |
| 1. | Urgency | 26.77 | 5.96 |  |  |  |  |  |  |
|  |  |  |  |  |  |  |  |  |  |
| 2. | Premeditation | 23.59 | 4.50 | .32** |  |  |  |  |  |
|  |  |  |  | [.20, .42] |  |  |  |  |  |
|  |  |  |  |  |  |  |  |  |  |
| 3. | Persistence | 20.40 | 4.41 | .40** | .26** |  |  |  |  |
|  |  |  |  | [.29, .50] | [.14, .37] |  |  |  |  |
|  |  |  |  |  |  |  |  |  |  |
| 4. | Sensation Seeking | 32.00 | 7.26 | -.00 | .32** | .01 |  |  |  |
|  |  |  |  | [-.12, .12] | [.21, .43] | [-.12, .13] |  |  |  |
|  | **MCQ** |  |  |  |  |  |  |  |  |
| 5. | log (*k*) | -2.28 | 0.75 | .14* | -.00 | .04 | -.01 |  |  |
|  |  |  |  | [.01, .25] | [-.12, .12] | [-.08, .16] | [-.14, .11] |  |  |
|  | **Substance use variables** | |  |  |  |  |  |  |  |
| 6. | Substance use frequency | 13.74 | 6.35 | .14** | .13* | .20** | .36** | .15** |  |
|  |  |  |  | [.01, .25] | [.01, .25] | [.08, .31] | [.25, .46] | [.03, .27] |  |
|  |  |  |  |  |  |  |  |  |  |
| 7. | Substance-related problems | 4.15 | 2.85 | .33** | .13* | .29** | .23** | .15* | .50** |
|  |  |  |  | [.22, .43] | [.01, .25] | [.17, .39] | [.11, .35] | [.03, .27] | [.41, .59] |
|  |  |  |  |  |  |  |  |  |  |

*Note.* *M* and *SD* represent mean and standard deviation, respectively. Values in square brackets indicate the 95% confidence interval. UPPS = Urgency Premeditation Perseverance Sensation Seeking Impulsive Behavior Scale, MCQ = Monetary Choice Questionnaire, *k* = delay discounting. * *p* < .05. ** *p* < .01.

**2.2 Partial correlations**

When controlling for substance use frequency, urgency (*r*(256)= .31, *p* < .001) and perseverance (*r*(256)= .22, *p* = .001) were significantly correlated with substance-related problems, while premeditation, sensation seeking and log(*k*) were not (all *p*s >.05).

**2.3 Relative contributions of impulsivity facets and delay discounting to substance use outcomes**

**Supplementary Table S 2**

*Hierarchical Multiple Regression Analyses with the UPPS Subscales and Delay Discounting Predicting Substance Use Frequency and Substance-Related Problems*

|  | Substance use frequency | | | | |  | Substance-related problems | | | | |
| --- | --- | --- | --- | --- | --- | --- | --- | --- | --- | --- | --- |
|  | ΔR^2^ | F | B | SE | β |  | ΔR^2^ | F | B | SE | β |
| *Step 1* | .083** | 23.05 |  |  |  |  | .017* | 4.432 |  |  |  |
| Male gender |  |  | 3.70 | 0.78 | .29** |  |  |  | 0.75 | 0.35 | .13* |
|  |  |  |  |  |  |  |  |  |  |  |  |
| *Step 2* | - | - |  |  |  |  | .239** | 81.26 |  |  |  |
| Male gender |  |  | - |  | - |  |  |  | -0.09 | 0.32 | -.02 |
| Substance use frequency |  |  | - |  | - |  |  |  | 0.23 | 0.03 | .51** |
|  |  |  |  |  |  |  |  |  |  |  |  |
| *Step 3 - UPPS* | .118** | 9.32 |  |  |  |  | .091** | 8.66 |  |  |  |
| Male gender |  |  | 2.29 | 0.84 | .18** |  |  |  | -0.04 | 0.34 | -.01 |
| Substance use frequency |  |  | - | - | - |  |  |  | 0.19 | 0.03 | .42** |
| Urgency |  |  | 0.13 | 0.07 | .13 |  |  |  | 0.13 | 0.03 | .25** |
| Premeditation |  |  | -0.04 | 0.09 | -.03 |  |  |  | -0.03 | 0.04 | -.06 |
| Perseverance |  |  | 0.20 | 0.09 | .14* |  |  |  | 0.07 | 0.04 | .11 |
| Sensation seeking |  |  | 0.26 | 0.06 | .30** |  |  |  | 0.04 | 0.02 | .10 |
|  |  |  |  |  |  |  |  |  |  |  |  |
| *Step 4 - MCQ* | .014* | 4.27 |  |  |  |  | .003 | 0.97 |  |  |  |
| Male gender |  |  | 2.04 | 0.85 | .16* |  |  |  | -0.01 | 0.35 | -.00 |
| Substance use frequency |  |  | - |  |  |  |  |  | 0.18 | 0.03 | .41** |
| Urgency |  |  | 0.11 | 0.07 | .10 |  |  |  | 0.12 | 0.03 | .25** |
| Premeditation |  |  | -0.04 | 0.09 | -.03 |  |  |  | -0.03 | 0.04 | -.06 |
| Perseverance |  |  | 0.21 | 0.09 | .14* |  |  |  | 0.07 | 0.04 | .12* |
| Sensation seeking |  |  | 0.27 | 0.06 | .31** |  |  |  | 0.04 | 0.02 | .11 |
| Log(*k*) |  |  | 1.00 | 0.49 | .12* |  |  |  | 0.20 | 0.20 | .05 |
|  |  |  |  |  |  |  |  |  |  |  |  |
| Total R^2^ | .215** | 11.4 |  |  |  |  | .349** | 19.08 |  |  |  |

*Note.* *ΔR^2^* = change in proportion of explained variance; B = unstandardized estimate; SE = standard error of B, β = standardized estimate. * *p* < .05. ** *p* < .01.
